# Diversity, Species Richness, and Community Composition of Wetland Birds in the Lowlands of Western Nepal

**DOI:** 10.1002/ece3.70538

**Published:** 2024-12-17

**Authors:** Siddha Raj Pant, Bishnu Prasad Bhattarai, Hem Sagar Baral, Tej Bahadur Thapa

**Affiliations:** ^1^ Central Department of Zoology, Institute of Science and Technology Tribhuvan University Kirtipur Nepal; ^2^ Kailali Multiple Campus, Far Western University Dhangadhi Nepal; ^3^ School of Veterinary, Environmental and Agriculture Sciences Charles Sturt University Albury New South Wales Australia

**Keywords:** conservation, ecosystems, habitats, protection, seasons, wetlands

## Abstract

Wetlands serve as crucial habitats for diverse bird species, playing a vital role in maintaining the ecosystem. Geographical location, climate, hydrology, and vegetation composition of wetlands determine the diversity of birds. This study investigated species richness, seasonal diversity, and composition of wetland birds in three prominent water bodies of western Nepal, namely Ghodaghodi, Rani, and Jokhar lakes, from 2021 to 2022 in summer and winter using the point count method. We observed 59 wetland bird species across three wetlands, where both the richness and diversity were significantly higher in the winter. Paired t‐tests revealed significant seasonal differences (*p* < 0.001) in diversity, species richness, and abundance at both Ghodaghodi and Rani lakes. The highest species richness was observed at Ghodaghodi (*n* = 58), reflecting its significance as a vital bird habitat. In contrast, diversity was higher in Rani Lake during both seasons (Winter *H* = 2.98, Summer *H* = 2.79). NMDS followed by ANISOM test also showed that bird community structure varies across lakes and seasons. Indicator species analysis showed that out of 59 species, 26 species are associated with one or different sites, whereas 18 of them are associated with the summer or winter season. We observed the Anatidae family with the highest relative diversity (RDI = 28.81%) and reported 1 globally vulnerable species and 7 globally near‐threatened species. While in Jokhar Lake, the abundance does not vary between the seasons. This study revealed substantial differences in bird species across seasons and lakes showing that each wetland offers a distinct ecological niche for bird species. The findings reinstate the importance of these wetlands as crucial habitats for the diversity of birds and species composition. This research contributes to the conservation and management of these ecosystems, aiding the protection of wetland birds in Nepal.

## Graphical abstract

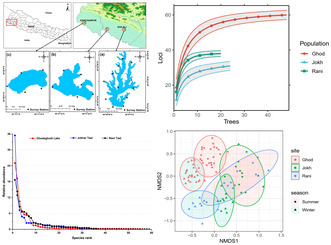

## 1 Introduction

Wetlands are fascinating ecosystems that provide a diverse range of habitats for various species of birds (Fournier et al. 2021; Gupta et al. 2020). These unique habitats, including open water, marshes, swamps, and bogs, support an incredible variety of bird life (Halls 1997). These wetlands are home to a range of wildlife including many bird species/types such as waders, waterfowl, shorebirds, and marsh birds (Grimmett et al. 2016; Khatiwada et al. 2021; Nayak and Bhushan 2022). Wetland birds have adapted to thrive in these watery landscapes, utilizing available resources and exhibiting specialized behaviors and physical characteristics. However, habitat requirements change seasonally due to their nesting, foraging, and breeding behaviors (Froneman et al. 2001).

Species richness and diversity of wetland birds are closely associated with vegetation structure and composition, wetland size, diversity and abundance of food resources, and water depth (Adhikari et al. 2022; Zakaria and Rajpar 2010). Unique characteristics of wetlands, like geographical location, climate, hydrology, and vegetation composition harbor diverse bird communities (Elliott, Igl, and Johnson 2020; Rajpar and Zakaria 2015). Despite such importance, wetlands are currently facing challenges such as habitat loss, pollution, and climate change both at the global level and locally in Nepal (Shin et al. 2022). Consequently, these threats have seriously altered the composition of wetland and wetland‐dependent birds across Nepal (Gautam et al. 2019; Thagunna, Subedi, and Koirala 2023).

The latest checklist reveals that there are 892 species of birds in Nepal (BCN, and DNPWC 2022). Among them, there are around 200 species of wetland and wetland‐associated birds in Nepal (Grimmett et al. 2016) and about 187 species can be found alone in the lowland Terai region (Baral and Inskipp 2020). These birds include large wading birds like cranes (Sharma et al. 2024) and storks (Bhattarai 2012) to small and medium‐sized species like gray‐headed canary flycatcher ( *Culicicapa ceylonensis* ), spangled drongo ( *Dicrurus hottentottus* ), and jungle babbler ( *Turdoides striata* ) (Dahal, McAlpine, and Maron 2014). However, growing anthropogenic expansion and intrusion into wetland habitats have altered wetland bird composition (Gautam et al. 2019; Thagunna, Subedi, and Koirala 2023). Despite such threats, only a few studies have been conducted to understand the overall composition of birds in wetland habitats. Most of the studies done in the lowland wetlands, especially in the west, are focused on single species like the sarus crane ( *Grus antigone* ) (Sharma et al. 2024), lesser adjutant ( *Leptoptilos javanicus* ) (Katuwal et al. 2022), Asian wooly neck stork ( *Ciconia episcopus* ) (Ghimire et al. 2023, 2021; Katuwal et al. 2020), and cotton pygmy‐goose ( *Nettapus coromandelianus* ) (Dangaura, Chaudhary, and Bhusal 2020).

Ghodaghodi, Rani, and Jokhar lakes in far‐western Nepal harbor diverse species of wetland birds. Though some studies have been done in these areas (Kafle 2005; Parajuli and Katuwal, 2015), studies particularly focusing on wetland bird communities and changes in seasonal patterns are notably lacking. With increasing threats to these wetlands, it is necessary to take serious measures to sustain the conservation of bird species in these wetland ecosystems. Understanding bird species composition based on wetland features is crucial to conserving birds. However, there are limited systematic and comparative studies done in these areas of Nepal to understand bird use of wetlands based on geographical, climatic, hydrological, and vegetation characteristics.

Our study aims to examine the species composition of wetland and wetland‐dependent birds across Ghodaghodi, Rani, and Jokhar lakes, and explore seasonal variations in bird composition within these wetland ecosystems. We aim to inform conservation and management efforts, thereby ensuring the preservation of these vital ecosystems in western Nepal. We hypothesize that Anatidae represents a highly abundant family of birds, and given the significance of these areas as wintering grounds for migratory birds, overall bird species composition is higher during winter than in summer.

## 2 Materials and Methods

### 2.1 Study Area

Study areas highlight the ecological significance of the Ghodaghodi, Jokhar, and Rani lakes, and explore three distinct low‐lying habitats that offer suitable habitats for both resident and migratory avifauna, playing a pivotal role in the western Nepal.

#### 2.1.1 Ghodaghodi Lake

**FIGURE 1:**
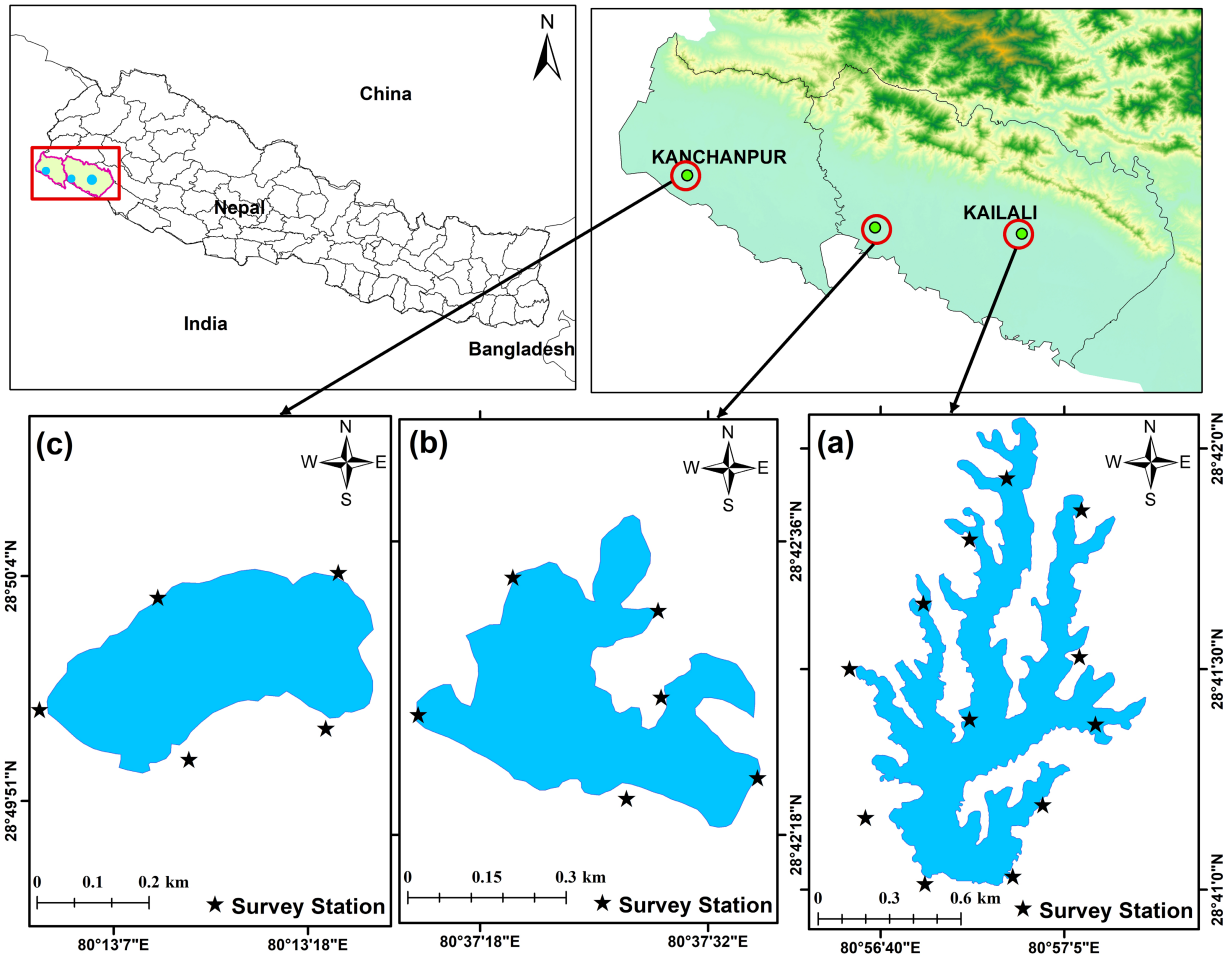
Map of study area showing their locations (a) Ghodaghodi, (b) Jokhar, and (c) Rani Lakes of Sudurpaschim Province, western Nepal.

Ghodaghodi Lake lies (28°41′20.39″ N, 80°56′54.66″  E) in Ghodaghodi Municipality of Kailali district, Sudurpaschim Province, Nepal situated at an elevation of 205 masl and having an area 2563 ha (Lamsal et al. 2014). Ghodaghodi Lake being rich in biodiversity is an important area for birds in Nepal and provides connectivity between the Terai plains and the Siwalik Hills (Figure 1a). The area was designated as a Ramsar site on 13 August 2003 and most recently on 11 March 2022, Sudurpaschim Province declared it as Nepal's first Bird sanctuary aiming to provide better management for sustainable use and promotion of biodiversity (Naunyal, Khadka, and Anderson 2023). Local residents depend on wetlands for fodder, aquatic macrophytes, livestock grazing, and fishing (Siwakoti and Karki 2009).

#### 2.1.2 Jokhar Lake

Jokhar Lake lies (28°42′23.90″ N, 80°37′21.48″ E), covering an area of 149 ha in the suburban area of Dhangadhi Sub‐Metropolitan City in Ward 7 of Kailali district, Sudurpaschim Province, Nepal (Figure 1b). The main source of water for this Lake is rainwater. Jokhar Lake is surrounded by forest on the North, East, and West and agriculture and human settlements on the South. The peripheral vegetation of this wetland is mixed deciduous Sal Forest dominated by *Shorea robusta* . Along with that, some other associated vegetations are *Terminalia alata, Syzigium cumini, Terminalia bellirica*, etc. The adjacent areas under human settlement are used for cultivation, homesteads, and livestock grazing including domestic livestock, for example, cattle, goats, and sheep.

#### 2.1.3 Rani Lake

Rani Lake is located (28°49′57.76″ N, 80°13′10.76″ E) inside the Shuklaphanta National Park, Kanchanpur, Nepal which covers a 65.45 ha area (Figure 1c). It is a protected site having rich floral and faunal diversity with various bird species. This is an oxbow lake formed by the shifting of the Chaudhar River. Only 13 km to the east of the Shuklaphanta National Park Office, this quaint little lake is located in the Singhpur area. Rani Lake is 900 m long and has a maximum depth of 3 m. The average temperature of this area is 25.9°C ranges from 14.3°C to 32°C. This area receives 1579 mm of precipitation annually (Adhikari et al. 2022).

This Lake is surrounded by dense Sal ( *Shorea robusta* ) forest, along with other tree species such as Kusum (*Scheleira oleosa*), Saaj (*Terminalia alata*), Jamun (*Syzygium cuminii*), and Bhellar (*Mallotus nudiflorus*) forest. Rani Lake is covered by elephant grass ( *Saccharum spontaneum* ), and Narenga ( *Narenga porphyrocoma* ) on the south, west, and east (Adhikari et al. 2022). This Lake is home to a variety of bird species, including the bronze‐winged Jacana and the yellow‐footed Green Pigeon, making it a haven of rich floral and faunal diversity. This Lake is also home to the mysterious skittering frog ( *Euphlyctis cyanophlyctis* ). Notably, the National Park surrounding the Lake is inhabited by different mammal species, including the Asian elephant ( *Elephas maximus* ), Swamp deer ( *Rucervus duvaucelii* ), Bengal tiger ( *Panthera tigris* ), and Leopard ( *Panthera pardus* ).

All three study areas offer suitable habitats for the Terai residents and migratory avifauna, which is pivotal in the province's ecosystem. Inside the wetlands, various plant species, including floating, submerged, and riparian plants, provide food, shelter, and nesting sites for a diverse avian population. These regions experience a tropical monsoon climate with an average annual precipitation varying from 1630 mm (observed in Tikapur, located 35 km southeast of the lake) to 1705 mm (recorded in Dhangadhi). Approximately 80%–85% of the total rainfall occurs during the monsoon season, spanning from mid‐June to late September. The monthly maximum temperatures in the area fluctuate between 21°C and 38°C, with corresponding minimum temperatures ranging from 6°C to 25°C at Tikapur (DHM 2019; Lamsal et al. 2014).

### 2.2 Methods

#### 2.2.1 Bird Survey

We conducted the survey at the study sites to assess the richness and diversity of wetland birds during two distinct seasons: the summer (March, April, and May) and the winter (December, January, and February) seasons in two consecutive years 2021 and 2022. For the study, we established a total of 23‐point count stations (12, 6, and 5 in Ghodaghodi Lake, Jokhar Lake, and Rani Lake, respectively) based on the geographic area of the wetlands. The stations were set up with a minimum inter‐sampling distance of 300 m to avoid double counting of birds (Rajpar and Zakaria 2015). The fixed‐point count method was employed for the study within a 50‐m radius from each fixed point to observe and identify birds accurately (Basaula et al. 2021; Bibby et al. 2000; Buckland, Marsden, and Green 2008; Shah 2021). All wetland birds observed or heard were recorded for 10 min (Escalante and Favero 2012; Mandal et al. 2021). During each season, point counts were conducted twice daily 7:00–10:00 AM and 3:00–6:00 PM when bird activity was highest. Binoculars (Wing 8 × 42) were used to aid bird observation and identification in the field (Grimmett et al. 2016). The taxonomic position (orders, families, genus, and species) of observed wetland birds were assigned from the “Birds of Nepal: An Official Checklist” (BCN 2023; DNPWC and BCN 2019). Globally threatened statuses were categorized according to the IUCN Red List of Threatened Species (IUCN 2023). Nationally threatened species were assigned as per the list of National Red List of Nepal's birds (Inskipp et al. 2016).

### 2.3 Data Analysis

To understand the composition and diversity of birds, we calculated a variety of indices including Shannon diversity, Pielou's evenness, Simpson diversity index, relative density index (RDI), distribution curve, and species accumulation curve.

The relative diversity index (RDI) was used to understand the composition of bird species families. The RDI was calculated as a proportion of species observed from each family to the total species observed. The formula for RDI is:

RDI=n/N×100

where, “*n*” is the number of bird species in a family, and “*N*” is the number of bird species across all the species (MacArthur 1960).

We calculated species richness as the total number of species observed in each season and each lake, and abundance as the total number of individuals observed. Species richness, abundance, diversity index, and species accumulation curves were calculated using the *vegan* package in R (Oksanen et al. 2013). Shannon–Wiener's diversity index (H′) and Pielou's evenness (J) were calculated to analyze bird species composition across the three lakes. The Shannon–Wiener index (H′) was calculated as:

$$H^{\prime }=-\mathrm{\Sigma Pi}\times \mathrm{LN}\;\left(\mathrm{Pi}\right)$$

where “Pi” is the proportion of each species in the sample and “LN (Pi)” is the natural logarithm of this proportion (Hutcheson 1970; Shannon 1948).

The evenness was calculated as:

$$J=H^{\prime }/\ln\left(S\right)$$

where “*H*′” is Shannon–Wiener's diversity index and “*S*” is species richness (Heip 1974).

A two‐way ANOVA was used to test for significant differences in species richness between lakes and seasons. Paired t‐test was used to evaluate the seasonal variations in species richness, abundance, and diversity indices between the studied lakes. A Whittaker plot was created by plotting species abundance against their ranks. The relative rank abundance curve (RAC) was employed to understand the species composition and diversity within the community (Avolio et al. 2019).

Non‐metric multidimensional scaling (NMDS) ordination using Bray‐Curtis dissimilarities, based on square‐root transformed species abundance data, was conducted to explore spatial and temporal variations in bird community structure. This analysis was performed using the *BiodiversityR* package, followed by the analysis of similarity (ANOSIM) test.

Indicator species analysis was performed using the *multipatt* function from the *indispecies* package. Detrended Correspondence Analysis (DCA) projected a gradient length of 3.45, indicating that Canonical Correspondence Analysis (CCA) was appropriate. The Monte Carlo permutation test with 9999 iterations was used to assess the significance of the ordination for environmental variables. A Venn diagram was prepared using the *VennDiagram* package to illustrate species overlap.

All analyses were performed in R Studio (R Core Team 2024, version 4.3.3, https://www.R‐project.org).

## 3 Results

### 3.1 Species Richness, Diversity, and Composition

**FIGURE 2:**
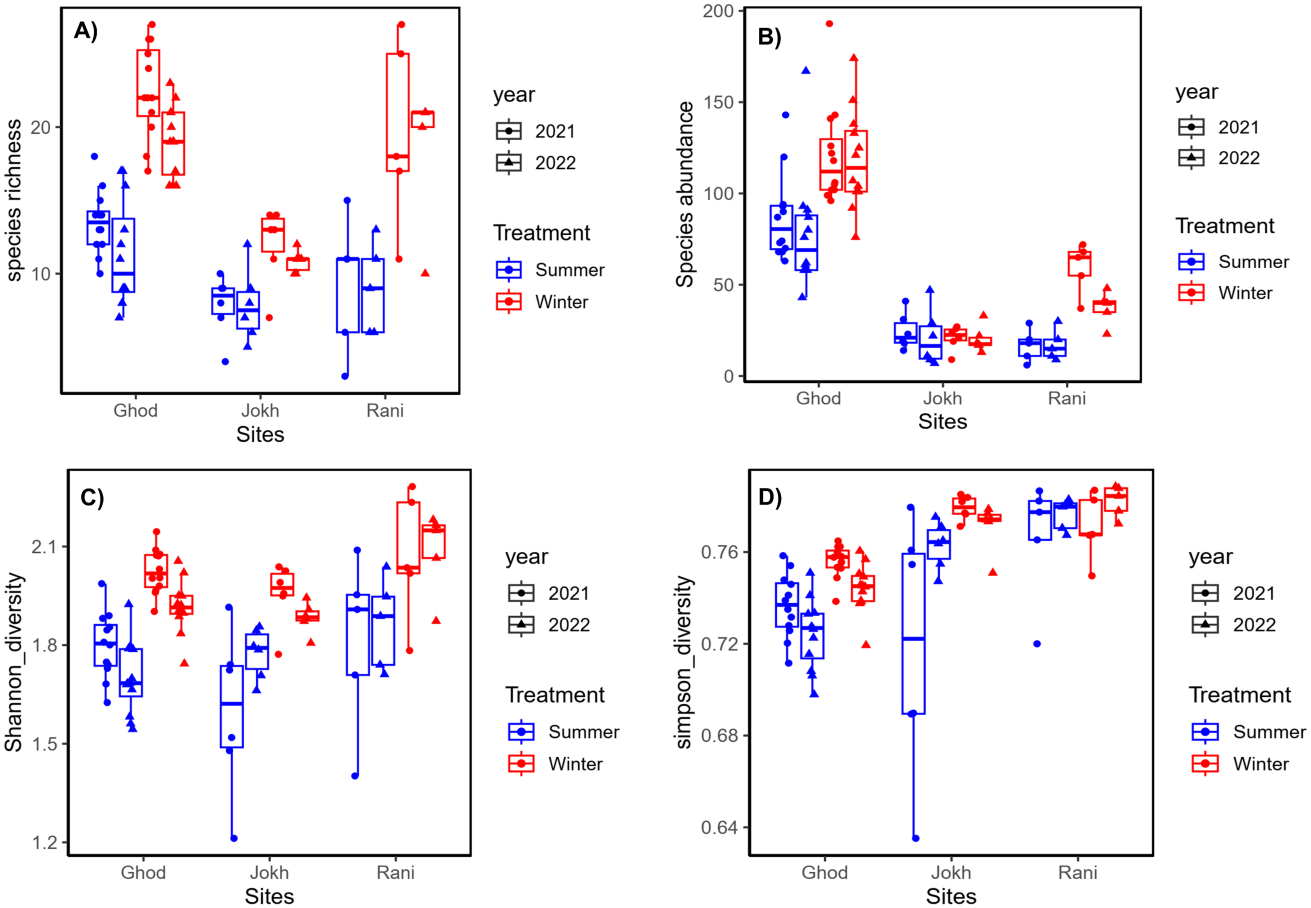
Boxplot showing bird species richness (A), abundance (B), Shannon (C) and Simpson (D) index of diversity in our study areas.

We recorded a total of 2277 individuals of birds belonging to 15 families, 39 genera, and 59 species in our study (Table S1). We observed a total species richness of 59 (Figure 2). We observed higher species richness in winter (*n* = 59) while the richness was 38 in summer with a significant difference between the seasons (*p* < 0.001). The species richness showed a significant difference in between the lakes (*p* < 0.001), where it was significantly higher for Ghodaghodi Lake in both seasons (winter = 58, summer = 32) as compared to others. We observed Rani Lake to be highly diverse in both the winter (*H*′ = 2.98) and summer season (*H*′ = 2.79). Also, Rani Lake showed the highest species evenness index during the summer (*J* = 0.60). However, species evenness was highest for Jokhar Lake in the winter (*J* = 0.58) (Table 1; Figure 2).

**TABLE 1:** Evenness of wetland birds in Ghodaghodi, Jokhar and Rani Lake.

Wetland	Pielou's evenness	Season
Ghodaghodi	0.36	Winter
	0.31	Summer
Jokhar	0.58	Winter
	0.38	Summer
Rani	0.51	Winter
	0.60	Summer

We observed the highest relative diversity values for the Anatidae family (RDI = 28.81%) followed by Ardeidae (16.95%), whereas the lowest RDI was observed for families Threskiornithidae, Anhingidae, Accipitridae, Pandionidae, and Podicipedidae which all accounted for the RDI 1.69% in the studied wetlands (Table 2). Our study reported 1 globally vulnerable ( *Aythya ferina* ) and 7 globally near‐threatened species (*Mareca falcata, Aythya nyroca, Leptoptilos javanicus, Ciconia episcopus, Anhinga melanogaster, Vanellus duvaucelii*, and *Icthyophaga ichthyaetus* ) (Table S1). This study also reported 2 nationally critically endangered species (*Mareca falcata*, *Icthyophaga ichthyaetus* ), 2 nationally endangered ( *Anas acuta* , *Sarkidiornis malanotos*), 6 nationally vulnerable species (*Nettapus coromandelianus, Aythya nyroca, Spatula querquedula, Anastomas oscitans, Leptoptilos javanicus, Hydrophasianus chirurgus*) and 7 nationally near‐threatened ( *Aythya ferina* , *Anas poecilorhyncha* , *Tadorna ferruginea, Ciconia episcopus, Anhinga melanogaster, Phalacrocorax carbo*) species of wetland birds in the study areas (Table 2; Table S1).

**TABLE 2:** Relative diversity index (RDI) of bird families observed in study sites.

S.N.	Avian family	Number of species recorded	RDI value
1	Anatidae	17	28.81
2	Podicipedidae	1	1.69
3	Rallidae	4	6.78
4	Ciconidae	3	5.08
5	Threskiornithidae	1	1.69
6	Ardeidae	10	16.95
7	Anhingidae	1	1.69
8	Phalacrocoracidae	2	3.39
9	Charadriidae	4	6.78
10	Jacanidae	2	3.39
11	Scolopacidae	3	5.08
12	Pandionidae	1	1.69
13	Accipitridae	1	1.69
14	Alcedinidae	4	6.78
15	Motacillidae	5	8.47
	Total(N)	59	100

**FIGURE 3:**
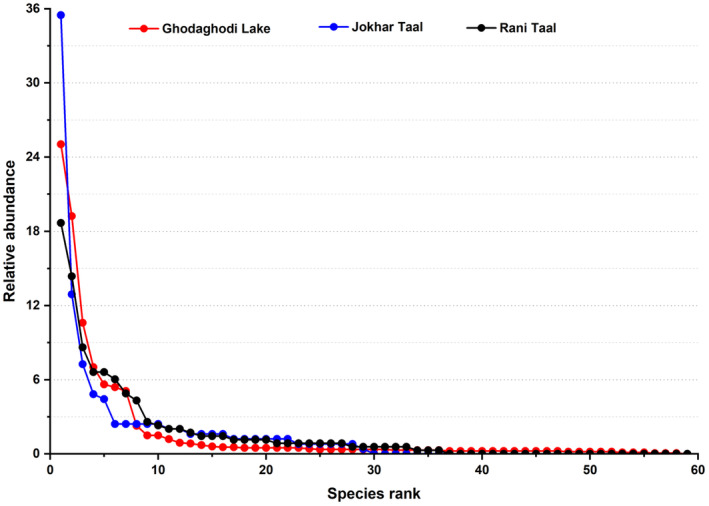
Relative rank abundance distribution curve of three different wetlands.

Analysis of the species abundance data revealed a consistent trend of low evenness across all three wetlands. High‐ranking species exhibited significantly higher abundances compared to low‐ranking species (Figure 3). This pattern shows the dominance of certain species within the wetland ecosystems. Furthermore, each wetland exhibited a distinct dominant species. *Dendrocygna javanica* was reported as the dominant species in Ghodaghodi Lake; similarly, *Aythya nyroca was in* Rani Lake, and *Bubulcus ibis* was in Jokhar Lake. These dominant species contributed significantly to the overall species composition and structure within their respective wetland.

**FIGURE 4:**
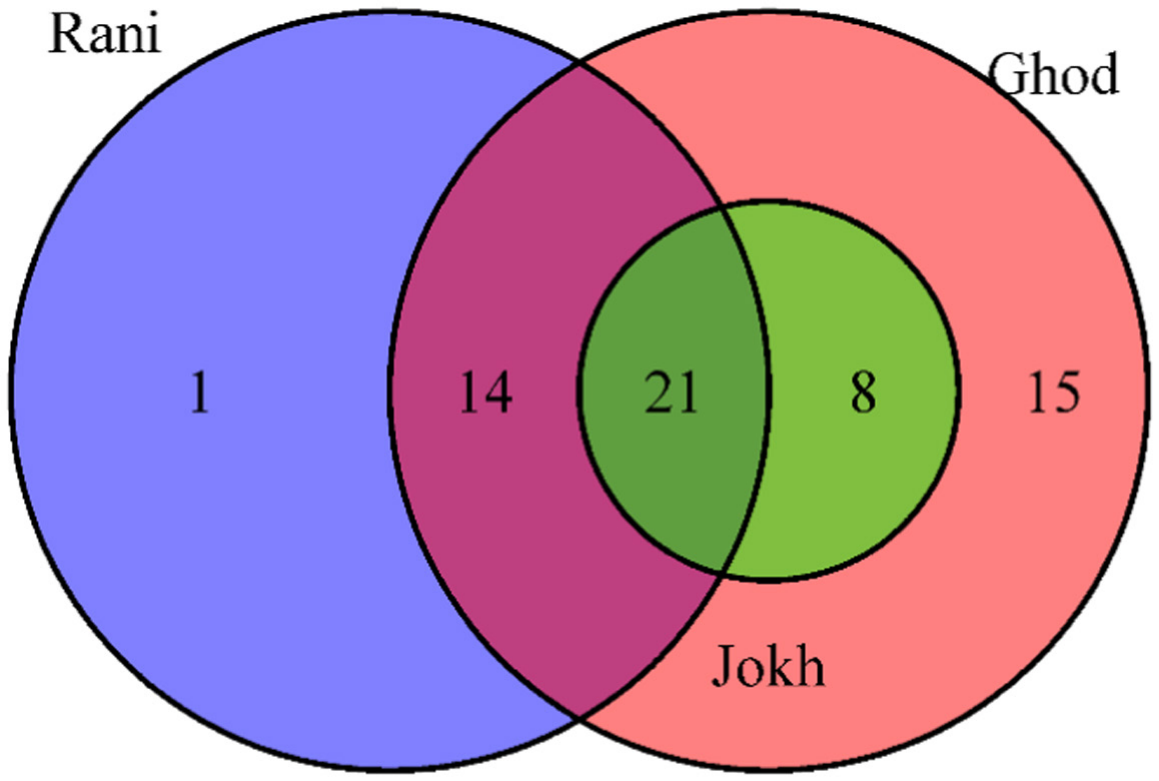
Venn diagram showing the sharing of wetland bird species between three lakes in Western Nepal. Lakes: Ghod‐ Ghodaghodi Lake, Jokh‐ Jokhar Lake, Rani‐ Rani Lake.

**FIGURE 5:**
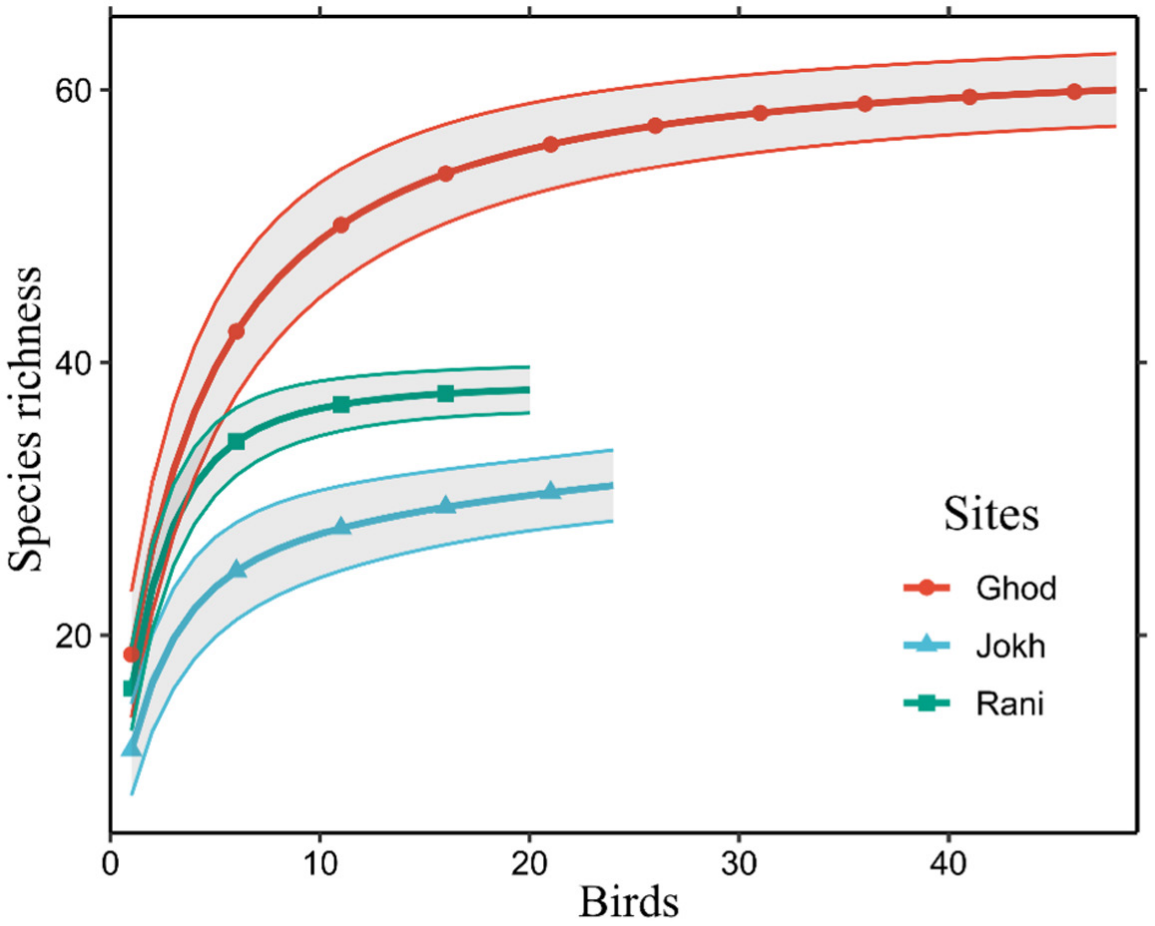
Species accumulation curve for wetland birds in three different lakes of western Nepal. Each line represents a different lake. Lakes: Ghod‐ Ghodaghodi Lake, Jokh‐ Jokhar Lake, Rani‐ Rani Lake.

Bird community composition exhibited significant seasonal variation across the three lakes, with higher diversity observed during the winter months (Figure 4). Paired *t*‐tests revealed significant differences (*p* < 0.001) between the summer and winter in Shannon diversity, Simpson diversity, species richness, and species abundance at both Ghodaghodi Lake and Rani Lake (Table S2). While a similar trend was observed at Jokhar, the differences in abundance were not significant. Furthermore, one‐way ANOVA analyses showed significant differences in diversity metrics between lakes for both seasons (*p* < 0.05 for all diversity indices) (Table 3; Figure 5).

**TABLE 3:** ANOVA of diversity indices and abundance between the three lakes.

	df	Winter		Summer	
		*f*‐value	*p*	f‐value	*p*
Shannon	2	63	0.003	2.45	0.09
Simpson	2	33.7	< 0.0001	8.64	< 0.001
Species richness	2	27.92	< 0.001	10.17	< 0.001
Abundance	2	99.72	< 0.001	47.14	< 0.001

### 3.2 Community Structure of Wetland Birds

**FIGURE 6:**
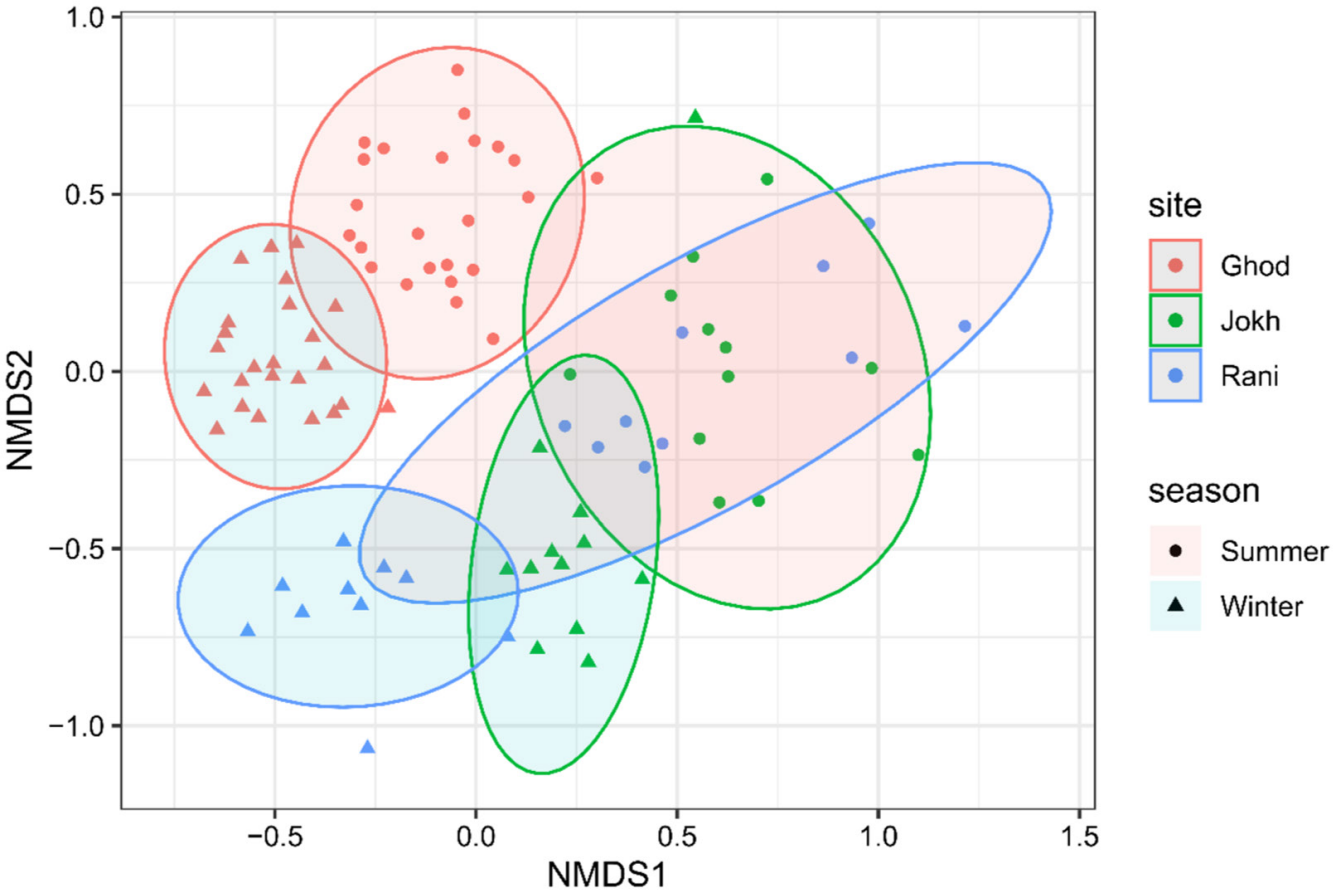
NMDS showing the influence of seasons and sites on the community composition of wetland birds in three different wetlands of western Nepal. Wetlands: Ghod‐ Ghodaghodi Lake, Jokh‐ Jokhar Lake, Rani‐ Rani Lake.

NMDS ordination resulted in a stress value of 0.2249, indicating a moderate fit of the ordination to the data (Figure 6). An ANOSIM test was performed to assess the significance of differences in community structure between lakes and seasons. ANOSIM test revealed a highly significant difference in community structure between the three study sites (*R* = 0.9158, *p* < 0.001). Similarly, ANOSIM test also reveals a significant difference in community structure between the summer and winter seasons (*R* = 0.1188, *p* < 0.001). NMDS plot also shows the clear demarcation between the community composition at different wetlands and between the seasons, with only a small overlap in the summer and winter seasons; however, the community composition of Rani Lake in the summer season shows a wide variation and more overlapped with the Jokhar Lake (Figure 6; Table 4).

**TABLE 4:** Result of DCA.

	DCA1	DCA2	DCA3	DCA4
Eigenvalues	0.4061	0.3178	0.104	0.091
Additive Eigenvalues	0.460	0.286	0.113	0.087
Decorana values	0.506	0.245	0.084	0.065
Axis length	3.465	2.952	2.194	1.297

**FIGURE 7:**
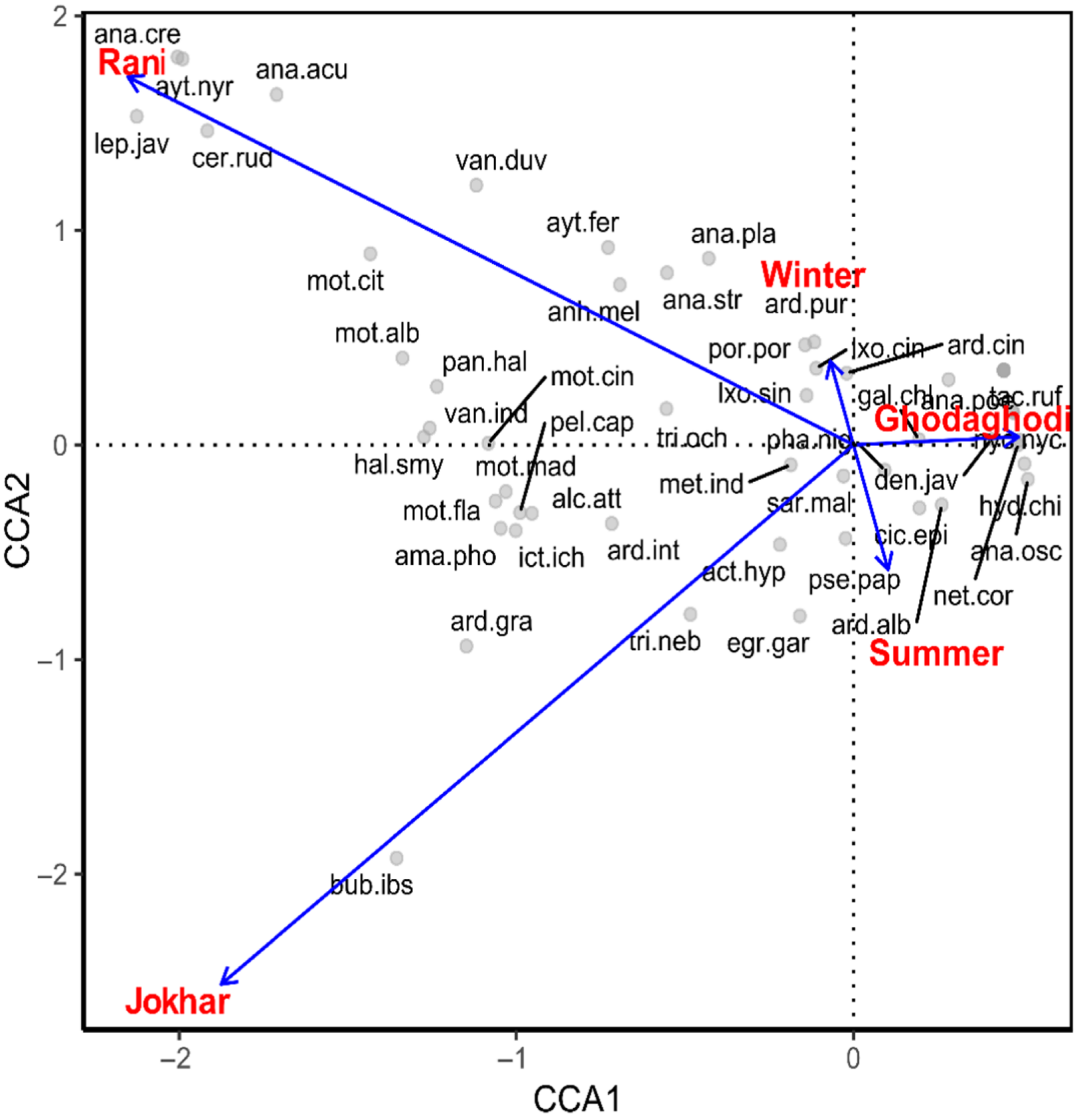
CCA—biplot showing the correlation between bird species and sites and season.

The species of birds are differently associated in studies habitat and between the seasons in this study. CCA (Figure 7) explained the 33.78% of the variance in the data. ANOVA of CCA shows that there is a significant impact of season and sites (*p* < 0.001) on the variance in the species composition (Table S4). Rani and Jokhar have major contribution in the variance in the bird's species associations. *Bubulcus ibis* shows more association with the Jokhar Lake. Similarly, bird species like *Anas crecca, Aythya nyroca, Anas acuta, Leptoptilos javanicus, and Ceryle rudis* show more association with Rani Lake. *Hydrophasianus chirurgus, Anastomas oscitans, and Nettapus coromandelianus* are associated with Ghodaghodi Lake.

### 3.3 Indicator Species Analysis

Indicator species analysis (A multilevel pattern analysis) was conducted to investigate the association of bird species with three distinct habitats (Ghod, Jokh, and Rani Wetlands). Out of 59 species, 26 were identified as significantly associated with at least one of the groups (alpha = 0.05). The analysis revealed a strong association of seven species with Ghodaghodi Lake (*Nettapus coromandelianus, Dendrocygna javanica, Nycticorax nycticorax, Phalacrocorax carbo, Fulica atra, Anas poecilorhyncha, Netta rufina*), one species with Jokhar Lake ( *Bubulcus ibis* ), and eight species with the Rani Lake (*Aythya nyroca, Ceryle rudis, Vanellus indicus, Motacilla citreola, Anas crecca, Leptoptilos javanicus, Anas acuta*, and *Vanellus duvaucelii* ). Additionally, two species exhibited significant associations with combined groups: *Pseudibis papillosa* and *Egretta garzetta* with Ghodaghodi and Jokhar, and *Gallinula chloropus* , *Ardea cinerea* , *Ardea purpurea* , *Mareca strepera*, *Anhinga melanogaster* , and *Porphyrio porphyrio* with Ghodaghodi and Rani Lake. Finally, two species were significantly associated with Jokhar and Rani lakes (*Halcyon smymimesis, Motacilla alba*) (Table S3).

Similarly, when exploring the seasonal variation in bird species assemblages, out of 59 species, 18 were significantly associated with one or the other season (alpha = 0.05). A single species, *Bubulcus ibis* , was identified as an indicator species of the summer season (IndVal = 0.772, *p* < 0.001). In contrast, 17 species displayed strong associations with the winter season. These findings suggest that the species assemblages and composition are influenced by habitat (Table S3).

## 4 Discussion

We found 59 birds species in the three lakes of western Nepal. The result indicates the variation of bird composition between these three lakes and two seasons. These lakes serve as feeding and breeding grounds, stopover sites, and wintering habitats for many bird species (Weller 1999) with their unique ecological features with rich avian diversity. This study shows that these lakes are important habitats for wetland birds boasting high richness and diversity across the seasons.

We observed Anatidae to be the dominant family which underscores their ecological significance in wetland habitats making them the most prevalent bird family with the highest RDI value in the studied sites. Our study findings are similar to the studies conducted in different parts of Haryana, India (Chopra and Sharma 2014; Kumar, Rai, and Gupta 2016; Kumar and Sharma 2019; Tak, Sati, and Rizvi 2010), where the researchers found Anatidae to be the largest wetland bird family. The reasons for their dominance may be due to their higher occupancy in the food chain (Li et al. 2021), migratory behavior, and wider distribution (Arzel, Elmberg, and Guillemain 2006; Mandal et al. 2021). The members of the Anatidae family are the indicators of a healthy wetland ecosystem (Amat and Green 2010). Among the three study sites, Ghodaghodi Lake has shown the largest number of Anatidae members, implying its healthier wetland ecosystem as well as a larger area of habitat. Therefore, this family is likely to benefit from the abundant food resources spread around a larger water body and suitable breeding conditions provided by these wetlands. Similarly, the presence of residential, migratory, and vagrant bird species indicates the diverse ecological roles played by these wetlands, supporting different communities and contributing to overall avian biodiversity (Amat and Green 2010; Nayak and Bhushan 2022). The abundant aquatic macrophytes create an ideal habitat for nesting and availability of food resources (Ali, Altaf, and Khan 2016; Mandal et al. 2021). For this study, Ardeidae has been the second most family with the largest species richness in these lakes. However, Ramesh et al. (2020) recorded Ardeidae as the most dominant family in Kalpakkam, Southeast coast of India.

The difference in the community structure of birds between the three lakes might be related to the differences in their size. Mostly, larger lakes provide larger space and suitable habitats for multiple species, thus supporting more species in a community (Chukwuka et al. 2022; Jambhekar, Suryawanshi, and Nagendra 2021). On the other hand, the smaller lakes might have limited habitat availability making them suitable for only a few selected species. Similarly, the differences in community composition between seasons might be due to the availability of resources. Generally, the composition changes as more resources are available during the summer season (Katuwal et al. 2022). However, these three lakes are the wintering ground as well as stopover sites for some bird species which can alter the community structure of the birds in this season.

We observed that the season is a determinant factor for the bird species composition across our study where species richness and diversity were higher during the winter season than in summer. This can be correlated to our study areas supporting the occurrence of the winter migratory birds. As evident from previous studies, these lakes function not only as winter breeding grounds for some species but also play a crucial role as stopover sites for long‐range migratory bird species (Adhikari et al. 2022; Khatri and Baral 2012; Weller 1999). This can also be due to reduced habitat quality during the summer as these wetlands may get partially dry which can reduce the species richness and diversity. Such decrease in species richness and diversity during summer can be seen in multiple studies across different wetlands of Nepal including Phewa Lake (Giri and Chalise 2008), Beeshhazari Lake (Adhikari, Bhattarai, and Dhakal 2018), Jagadishpur Reservoir (Thapa and Saud 2012) and Ramaroshan (Tachamo‐Shah et al. 2023).

Our study suggests a higher richness of wetland birds in Ghodaghodi Lake in comparison to others. This might be due to the larger area of the lake in comparison to others as a large area often has larger habitat thus supporting higher species richness (Maseko et al. 2020). However, in such a comparison between wetland areas, it may be necessary to consider factors that may play a role in species richness and diversity. Ghodaghodi complex shows a combination of marshes, shallow lakes, and rivers, providing a wide range of habitats for wetland birds due to large, interconnected lakes (Lamsal et al. 2014), supporting both residential and migratory birds arriving as far as from Siberia and Europe (Naunyal, Khadka, and Anderson 2023). The abundance of vegetation and aquatic resources supports a high level of productivity, resulting in a rich food supply, and providing breeding grounds for the resident and migratory birds. The smaller habitat area and wetland size of Rani Lake and Jokhar Lake compared to Ghodaghodi Lake may have implications for the relatively lower abundance and diversity of bird species in these areas (Naunyal, Khadka, and Anderson 2023).

Despite having a smaller size in comparison to the other two, Rani Lake boasts a high diversity of wetland birds across both seasons in our study sites. This might be due to the lake being relatively away from the settlement and in the midst of natural habitat. The lake is situated inside the Shuklaphanta National Park which may reduce potential threats to the wetland birds residing in the area and thus support higher diversity. This can be further confirmed by our finding which shows relatively similar evenness of bird species in the area. Natural and undisturbed habitats often provide better refuge for bird species, thus supporting higher diversity in comparison to disturbed areas (Arias‐Sosa, Salamanca‐Reyes, and Ramos‐Montaño 2021; Beaudry et al. 2013). Similar patterns of diversity can be found across multiple studies including Chawaka et al. (2017) and Ntongani and Andrew (2013). However, other studies suggest otherwise, indicating a positive or significant association between human disturbance and bird diversity (Mereta et al. 2021). Thus, the relationship between human disturbance and bird diversity might be context‐dependent.

Similarly, the species evenness was higher in Jokhar Lake in the winter which could be attributed to the lower richness of the area as richness often shows a negative association with evenness (Stirling and Wilsey 2001; Symonds and Johnson 2008). The area with lower species richness may result in proper resource partitioning between the species rather than competition, thus making the distribution even. Further, Jokhar Lake is subject to relatively smaller and has higher human disturbance which may result in increased abundance (BCN, and DNPWC 2022). As a result of reduced competition, there might be more even distribution than less disturbed habitats. Similarly, in highly disturbed areas, the richness inclines mostly towards tolerant species (Tryjanowski et al. 2020) and due to their ability to co‐exist with human disturbances, they may assist in increased species evenness.

Ghodaghodi Lake, Rani Lake, and Jokhar Lake are known for their ecological importance and support a diverse array of bird species. However, despite their high species richness, it is seen that Jokhar and Rani Lake have low species evenness as compared to the Ghodaghodi Lake. In the case of Ghodaghodi, Rani Lake, and Jokhar Lake, the RACs exhibit a characteristic pattern of low species evenness. This pattern suggests that certain species dominate the wetland bird communities, while others are less abundant. The curve tends to have a steep slope, indicating a few dominant species with higher abundances, followed by a rapid decline in abundance for the remaining species.

## 5 Conclusions

This study underscores the significance of understanding the abundance and seasonal diversity of wetland birds in the lowland areas of Sudurpaschim Province, Nepal, providing valuable insights into the dynamics of species composition across the seasons and lakes. The variations in species richness, diversity, and evenness between seasons and lakes highlight the potential influence of seasonality, migration patterns, and habitat dynamics on the composition of wetland birds. These findings contribute to our understanding of the ecological dynamics of wetland birds and can inform conservation efforts aimed at preserving the biodiversity and functioning of these important ecosystems. However, long‐term monitoring and continued assessment of species' status are crucial to understanding the prevailing scenario and to ensure the sustainable functioning of these wetlands for the conservation of wetland birds.

## Author Contributions

**Siddha Raj Pant:** conceptualization (lead), data curation (lead), formal analysis (lead), methodology (lead), software (lead), writing – original draft (lead). **Bishnu Prasad Bhattarai:** formal analysis (lead), methodology (lead), software (lead), writing – review and editing (lead). **Hem Sagar Baral:** supervision (equal), validation (lead), visualization (lead), writing – review and editing (equal). **Tej Bahadur Thapa:** conceptualization (lead), methodology (lead), supervision (lead), validation (lead), visualization (lead), writing – review and editing (lead).

## Ethics Statement

We did not capture the species; we collected the data from the concerned authorities, and we got permission to visit the sites. So do not require any ethical clearance.

## Conflicts of Interest

The authors declare no conflicts of interest.

## Supporting information


**Table S1.** List of wetland birds recorded in Ghodaghodi Lake (G), Jokhar Lake (J) and Rani Lake (R) from January 2021 to December 2022 along with their respective taxonomic positions.
**Table S2.** Paired *t*‐test for each lake for summer and winter seasons.
**Table S3.** Indicator species analysis for season and sites.
**Table S4.** Permutation test for CCA with 9999 iterations to assess the significance of the ordination.

## Data Availability

Data and codes used in this paper are available through dryad (DOI: 10.5061/dryad.r2280gbmw) and zenodo (https://zenodo.org/records/13282833). Reviewer link https://datadryad.org/stash/share/zN5jQBOmEbU8IGEdbaXe0B1onbdj3DhW51j6NNUCrtk.
