# Supplementary material for: Diversity, Species Richness, and Community Composition of Wetland Birds in the Lowlands of Western Nepal
Source: Ecol Evol. 2024 Dec 17;14(12):e70538. doi: 10.1002/ece3.70538 (PMC11650748; doi:10.1002/ece3.70538)
Supplement: Supplementary file 1 — Table S1. List of wetland birds recorded in Ghodaghodi Lake (G), Jokhar Lake (J) and Rani Lake (R) from January 2021 to December 2022 along with their respective taxonomic positions. Table S2. Paired t‐test for each lake for summer and winter seasons. Table S3. Indicator species analysis for season and sites. Table S4. Permutation test for CCA with 9999 iterations to assess the significance of the ordination. [file ECE3-14-e70538-s001.zip › Pant et al 2024-Supplementary Tables.docx]

**Supplementary Table 1 (Table A1).**

List of Wetland birds recorded in Ghodaghodi Lake (G), Jokhar Taal (J) and Rani Taal (R) from January 2021 to December 2022 along with their respective Taxonomic positions.

| S.N. | Common Name | Scientific Name | Codes used in the analysis | Order | Family | Sites | IUCN Status | |
| --- | --- | --- | --- | --- | --- | --- | --- | --- |
|  |  |  |  |  |  |  | GTS | NTS |
| 1 | Lesser whistling- duck | *Dendrocygna javanica* | den.jav | Anseriformes | Anatidae | G, J, R | LC | LC |
| 2 | Ruddy Shelduck | *Tadorna ferruginea* | tad.fer | Anseriformes | Anatidae | G | LC | NT |
| 3 | African Comb Duck | *Sarkidiornis malanotos* | sar.mal | Anseriformes | Anatidae | G, J | LC | LC |
| 4 | Cotton Pygmy- goose | *Nettapus coromandelianus* | net.cor | Anseriformes | Anatidae | G, J | LC | VU |
| 5 | Red-crested Pochard | *Netta rufina* | net.ruf | Anseriformes | Anatidae | G | LC | LC |
| 6 | Common Pochard | *Aythya ferina* | ayt.fer | Anseriformes | Anatidae | G, R | VU | NT |
| 7 | Ferruginous Duck | *Aythya nyroca* | ayt.nyr | Anseriformes | Anatidae | G, R | NT | VU |
| 8 | Tufted Duck | *Aythya fuligula* | ayt.ful | Anseriformes | Anatidae | G | LC | LC |
| 9 | Garganey | *Spatula querquedula* | spa.que | Anseriformes | Anatidae | G | LC | VU |
| 10 | Northern Shoveler | *Spatula clypeata* | spa.cly | Anseriformes | Anatidae | G | LC | LC |
| 11 | Falcated Duck | *Mareca falcate* | mar.fal | Anseriformes | Anatidae | G | NT | CR |
| 12 | Gadwall | *Mareca strepera* | mar.str | Anseriformes | Anatidae | G, J, R | LC | LC |
| 13 | Eurasian Wigeon | *Mareca Penelope* | mar.pen | Anseriformes | Anatidae | G | LC | LC |
| 14 | Indian Spot-billed Duck | *Anas poecilorhyncha* | ana.poe | Anseriformes | Anatidae | G | LC | NT |
| 15 | Mallard | *Anas platyrhynchos* | ana.pla | Anseriformes | Anatidae | G, R | LC | LC |
| 16 | Northern Pintail | *Anas acuta* | ana.acu | Anseriformes | Anatidae | G, R | LC | EN |
| 17 | Common Teal | *Anas crecca* | ana.cre | Anseriformes | Anatidae | G, R | LC | LC |
| 18 | Little Grebe | *Tachybaptus ruficollis* | tac.ruf | Podicipediformes | Podicipedidae | G | LC | LC |
| 19 | White-breasted Waterhen | *Amaurornis phoenicurus* | ama.pho | Gruiformes | Rallidae | G, J. R | LC | LC |
| 20 | Purple Swamphen | *Porphyrio porphyrio* | por.por | Gruiformes | Rallidae | G, R | LC | LC |
| 21 | Common Moorhen | *Gallinula chloropus* | gal.chl | Gruiformes | Rallidae | G, J, R | LC | LC |
| 22 | Common Coot | *Fulica atra* | ful.atr | Gruiformes | Rallidae | G | LC | LC |
| 23 | Lesser Adjutant | *Leptoptilos javanicus* | lep.jav | Ciconiiformes | Ciconiidae | R | NT | VU |
| 24 | Asian Openbill | *Anastomas oscitans* | ana.osc | Ciconiiformes | Ciconidae | G | LC | VU |
| 25 | Asian Woollyneck | *Ciconia episcopus* | cic.epi | Ciconiiformes | Ciconiidae | G, J | NT | NT |
| 26 | Red-naped Ibis | *Pseudibis papillosa* | pse.pap | Pelecaniformes | Threskiornithidae | G, J | LC | LC |
| 27 | Yellow Bittern | *Ixobrychus sinensis* | lxo.sin | Pelecaniformes | Ardeidae | G, R | LC | LC |
| 28 | Cinnamon Bittern | *Ixobrychus cinnamomeus* | lxo.cin | Pelecaniformes | Ardeidae | G, R | LC | LC |
| 29 | Black-crowned Night –heron | *Nycticorax nycticorax* | nyc.nyc | Pelecaniformes | Ardeidae | G | LC | LC |
| 30 | Indian Pond- heron | *Ardeola grayii* | ard.gra | Pelecaniformes | Ardeidae | G, J, R | LC | LC |
| 31 | Cattle Egret | *Bubulcus ibis* | bub.ibs | Pelecaniformes | Ardeidae | G, J, R | LC | LC |
| 32 | Grey Heron | *Ardea cinerea* | ard.cin | Pelecaniformes | Ardeidae | G R | LC | LC |
| 33 | Purple Heron | *Ardea purpurea* | ard.pur | Pelecaniformes | Ardeidae | G, R | LC | LC |
| 34 | Great White Egret | *Ardea alba* | ard.alb | Pelecaniformes | Ardeidae | G, J | LC | LC |
| 35 | Intermediate Egret | *Ardea intermedia* | ard.int | Pelecaniformes | Ardeidae | G, J, R | LC | LC |
| 36 | Little Egret | *Egretta garzetta* | egr.gar | Pelecaniformes | Ardeidae | G, J | LC | LC |
| 37 | Little Cormorant | *Microcarbo niger* | mic.nig | Suliformes | Phalacrocoracidae | G, J, R | LC | LC |
| 38 | Great Cormorant | *Phalacrocorax carbo* | pha.car | Suliformes | Phalacrocoracidae | G, R | LC | NT |
| 39 | Oriental Darter | *Anhinga melanogaster* | anh.mel | Suliformes | Anhingidae | G, R | NT | NT |
| 40 | Little Ringed Plover | *Charadrius dubius* | cha.dub | Charadriiformes | Charadriidae | G | LC | LC |
| 41 | River Lapwing | *Vanellus duvaucelii* | van.duv | Charadriiformes | Charadriidae | G, R | NT | NT |
| 42 | Red-wattled Lapwing | *Vanellus indicus* | van.ind | Charadriiformes | Charadriidae | G, J, R | LC | LC |
| 43 | Pheasant-tailed Jacana | *Hydrophasianus chirurgus* | hyd.chi | Charadriiformes | Jacanidae | G | LC | VU |
| 44 | Bronze-winged Jacana | *Metopidius indicus* | met.ind | Charadriiformes | Jacanidae | G, J, R | LC | LC |
| 45 | Common Sandpiper | *Actitis hypoleucos* | act.hyp | Charadriiformes | Scolopacidae | G, J | LC | LC |
| 46 | Green Sandpiper | *Tringa ochropus* | tri.och | Charadriiformes | Scolopacidae | G, J, R | LC | LC |
| 47 | Common Greenshank | *Tringa nebularia* | tri.neb | Charadriiformes | Scolopacidae | G, J | LC | LC |
| 48 | Marsh Sandpiper | *Tringa stagnatilis* | tri.sta | Charadriiformes | Scolopacidae | G | LC | LC |
| 49 | Osprey | *Pandion haliaetus* | pan.hal | Accipitriformes | Pandionidae | G, J, R | LC | LC |
| 50 | Grey-headed Fish- eagle | *Icthyophaga ichthyaetus* | ict.ich | Accipitriformes | Accipitridae | G, J, R | NT | CR |
| 51 | Common Kingfisher | *Alcedo atthis* | alc.att | Coraciiformes | Alcedinidae | G, J, R | LC | LC |
| 52 | Pied Kingfisher | *Ceryle rudis* | cer.rud | Coraciiformes | Alcedinidae | G, R | LC | LC |
| 53 | Stork billed kingfisher | *Pelargopsis capensis* | pel.cap | Coraciiformes | Alcedinidae | G, J, R | LC | LC |
| 54 | White-breasted Kingfisher | *Halcyon smymimesis* | hal.smy | Coraciiformes | Alcedinidae | G, J, R | LC | LC |
| 55 | Western Yellow Wagtail | *Motacilla flava* | mot.fla | Passeriformes | Motacillidae | G, J, R | LC | LC |
| 56 | Grey Wagtail | *Motacilla cinerea* | mot.cin | Passeriformes | Motacillidae | G, J, R | LC | LC |
| 57 | Citrine Wagtail | *Motacilla citreola* | mot.cit | Passeriformes | Motacillidae | G, J, R | LC | LC |
| 58 | White-browed Wagtail | *Motacilla maderaspatensis* | mot.mad | Passeriformes | Motacillidae | G, J, R | LC | LC |
| 59 | White Wagtail | *Motacilla alba* | mot.alb | Passeriformes | Motacillidae | G, J, R | LC | LC |

Note: IUCN=International Union for Conservation of Nature, VU= Vulnerable, NT= Near Threatened and LC= Least Concern, CR=Critically Endangered, GTS= Globally Threatened Status, NTS= Nationally Threatened Status

**Supplementary Table 2 (Table A2).**

**Paired t-test for each lake for summer and winter seasons**

| Ghodaghodi | | | |
| --- | --- | --- | --- |
|  | t-value | Df | p-value |
| Shannon | -9.603 | 23 | <0.001 |
| Simpson | -7.592 | 23 | <0.001 |
| species richness | -9.4492 | 23 | <0.001 |
| Abundance | -11.161 | 23 | <0.001 |
| Rani | | | |
|  | t-value | Df | p-value |
| Shannon | -8.797 | 9 | <0.001 |
| Simpson | -1.646 | 9 | <0.001 |
| species richness | -9.12 | 23 | <0.001 |
| Abundance | -7.086 | 9 | <0.001 |
| Jokhar | | | |
|  | t-value | Df | p-value |
| Shannon | -3.204 | 11 | 0.008 |
| Simpson | -2.541 | 11 | 0.027 |
| species richness | -4.019 | 11 | 0.002 |
| Abundance | 0.589 | 11 | 0.567 |

**Supplementary Table 3 (Table A3).**

**Indicator species analysis for season and sites:**

| Winter | | | |
| --- | --- | --- | --- |
| **Speices** | **stat** | **p.value** |  |
| mot.cit | 0.821 | 0.0001 | *** |
| mot.cin | 0.737 | 0.0001 | *** |
| mot.alb | 0.737 | 0.0001 | *** |
| ful.atr | 0.722 | 0.0001 | *** |
| ana.str | 0.711 | 0.0001 | *** |
| pha.car | 0.645 | 0.0015 | ** |
| pan.hal | 0.626 | 0.0001 | *** |
| mot.fla | 0.608 | 0.0002 | *** |
| ard.pur | 0.569 | 0.0028 | ** |
| ana.pla | 0.532 | 0.0001 | *** |
| ayt.nyr | 0.511 | 0.0002 | *** |
| ana.cre | 0.489 | 0.0008 | *** |
| ana.acu | 0.466 | 0.0008 | *** |
| act.hyp | 0.432 | 0.0208 | * |
| net.ruf | 0.417 | 0.0049 | ** |
| Summer | | | |
| bub.ibs | 0.772 | 0.0001 | *** |
| Ghodaghodi | | | |
| net.cor | 0.997 | 0.0001 | *** |
| den.jav | 0.962 | 0.0001 | *** |
| nyc.nyc | 0.777 | 0.0001 | *** |
| pha.car | 0.739 | 0.0002 | *** |
| ful.atr | 0.707 | 0.0001 | *** |
| ana.poe | 0.661 | 0.0001 | *** |
| net.ruf | 0.408 | 0.025 | * |
| Jokhar | | | |
| bub.ibs | 0.695 | 0.0152 | * |
| Rani | | | |
| ayt.nyr | 0.66 | 0.0001 | *** |
| cer.rud | 0.658 | 0.0001 | *** |
| van.ind | 0.647 | 0.0004 | *** |
| mot.cit | 0.596 | 0.0335 | * |
| ana.cre | 0.583 | 0.0002 | *** |
| lep.jav | 0.548 | 0.0003 | *** |
| ana.acu | 0.479 | 0.005 | ** |
| van.duv | 0.396 | 0.0159 | * |
| ghod+jok | | | |
| pse.pap | 0.612 | 0.0023 | ** |
| egr.gar | 0.553 | 0.0097 | ** |
| Ghod+rani | | | |
| gal.chl | 0.93 | 0.0001 | *** |
| ard.cin | 0.642 | 0.0006 | *** |
| ard.pur | 0.642 | 0.0006 | *** |
| ana.str | 0.582 | 0.0061 | ** |
| anh.mel | 0.529 | 0.0064 | ** |
| por.por | 0.514 | 0.0085 | ** |
| Johk+rani | | | |
| hal.smy | 0.677 | 0.0014 | ** |
| mot.alb | 0.571 | 0.0069 | ** |

Table A3: Permutation test for cca with 9999 iterations to assess the significance of the ordination.

|  | Df | Chisquare | F | Pr(>F) |
| --- | --- | --- | --- | --- |
| Sites | 2 | 0.68 | 18.94 | <0.001 |
| Season | 1 | 0.105 | 5.86 | <0.001 |
| Residual | 88 | 1.58 |  |  |
| Signif. codes: 0 ‘***’ 0.001 ‘**’ 0.01 ‘*’ 0.05 ‘.’ 0.1 ‘ ’ 1 | | | | |
